# The rumen-derived *Lact. mucosae* LLK-XR1 exhibited greater free gossypol degradation capacity during solid-state fermentation of cottonseed meal and probiotic potential

**DOI:** 10.1186/s12866-023-03156-6

**Published:** 2024-01-05

**Authors:** Liangkang Lv, Fengliang Xiong, Yingyi Liu, Shiteng Pei, Shanshan He, Shengli Li, Hongjian Yang

**Affiliations:** State Key Laboratory of Animal Nutrition, College of Animal Science and Technology, China Agricultural University, Beijing, 100193 China

**Keywords:** Rumen-derived bacteria, *Lact. mucosae* strains, Detoxification, Gossypol, Fermentation

## Abstract

**Background:**

This study aimed to isolate the rumen-derived bacteria with the ability to degrade free gossypol (FG), and to evaluate the probiotic potential in vitro for ensuring safe utilization.

**Methods:**

The strains were anaerobically isolated from fresh rumen fluid of sheep with long-term fed cottonseed meal (CSM) with the screening agar medium containing gossypol as the sole carbon source. Afterwards, the isolated strain incubated with CSM was subjected to the determination of the FG degradation and in vitro evaluation of probiotic characteristics.

**Results:**

The target strain labeled *Lact. mucosae* LLK-XR1 [Accession number: OQ652016.1] was obtained, and its growth on MRS Liquid medium exhibited degradation efficiency of FG up to 69.5% which was significantly greater than its growth on Man-Rogosa-Sharpe medium with glucose free for 24 h (*p* < 0.01). Meanwhile, LLK-XR1 showed 40.652% degradation rate of FG for unautoclaved, non-pulverized, and no additional nutrients supplementation CSM. Furthermore, LLK-XR1 presented good survivability at pH 3.0 (above 88.6%), and 0.3% bile (78.5%). LLK-XR1 showed sensitivity to broad-spectrum antibiotics except Sulfamethoxazole, Ciprofloxacin and Gentamycin and significantly inhibited *E. coli* CICC 10,899, *Staph. aureus* CICC 21,600, and *Salmonella. Typhimurium* CICC 21,483. LLK-XR1 demonstrated good cell surface hydrophobicity and auto-aggregation ability.

**Conclusions:**

Taken together, this study for the first time noted that rumen-originated *Lact. mucosae* LLK-XR1 with probiotic properties exhibited substantial FG degradation capacity when it was applied to the solid-state fermentation of CSM.

**Supplementary Information:**

The online version contains supplementary material available at 10.1186/s12866-023-03156-6.

## Background

Gossypol, a natural phenolic compound derived from cotton plants (Gossypium spp.), primarily exists in two forms, free gossypol (FG) and bound gossypol (BG), within the roots, stems, leaves, and seeds of cotton plants [1]. Remarkably, the BG exhibits non-toxicity properties due to its limited absorption in the digestive tract, while a fraction of gossypol in the bound form may undergo subsequent release as FG during animal digestion [2, 3]. Meanwhile, the toxic nature of FG towards livestock has been widely acknowledged, its content of cotton by-products represents a main constraining factor in their utilization as feed sources, and the adverse effects of toxicity are manifested in animal performance, reproductive disorders, and immunotoxicity [1, 4, 5]. For instance, long-term feeding of 50% cotton stalk significantly reduces the weight gain performance of sheep and causes inflammatory damage to their liver and kidneys [6]. Feeding cottonseed to beef cattle of 416 ± 9.7 kg has a negative impact on dry matter intake, apparent digestibility, and ruminal fermentation characteristics [7]. Boars experiencing gossypol-induced stress exhibited a significant increase in the percentage of spermatozoa displaying tail abnormalities in the semen, coupled with a reduction in the number of spermatogonia in their seminiferous tubules [8]. Various methods of gossypol degradation were gradually being developed and investigated, but achieving optimal solutions for efficient gossypol degradation without compromising cotton by-products nutritional value and process costs remains challenging [9].

Therefore, we attempted to identify some key factors that may be effective in gossypol degradation with the help of keyword co-occurrence network analysis by VOSviewer software (Leiden University, Leiden, Netherlands) [10]. A high-frequency keyword analysis can be employed to identify prominent research areas and focal points within the field of the target investigation, highlighting the prevailing trends and potential advantageous factors in research [11]. This detailed content can be found in the Supplementary Materials. Overall, it is noteworthy that ruminants have the relatively higher tolerance to gossypol compared to monogastric animals, despite gossypol still remains to have adverse effects on ruminants [3, 12]. Meanwhile, rumen-originated microbial fermentation presented the potentially promising method for the detoxification of gossypol, and exhibited the potential to concurrently address the above-associated concerns [13–15].

The results of the summary and analysis of the previous studies suggested that the utilization of ruminal microorganisms holds promise for the degradation of FG in animal feed. Accordingly, we hypothesized that the existence of rumen-derived microbes possesses the capability to degrade gossypol. In the current experiment, our objective was to isolate and purify endogenous rumen microorganisms capable of efficiently degrading gossypol. Furthermore, to assess the ability of FG degradation and the probiotic properties of target strains.

## Methods

### Preparation of rumen fluid inoculum and culture medium

Fresh rumen fluid was collected from sheep at Xuntian Husbandry Co., Ltd, Hebei, China. The rumen fluid was directly aspirated from thirty-five rumen-cannulated Dorper × Hu hybrid sheep, with an average age of 4.5 months and an average body weight of 38.32 ± 0.94 kg. The sheep were fed the base total mixed ration, based on rice straw, corn and cotton meal throughout the entire study period. For each round of strain screening, approximately 250 mL of rumen fluid from each of 3 sheep before the morning feeding, underwent filtration using 4 layers of sterile gauze. The filtered fluid was then mixed, pooled, and sealed within a preheated thermos flask. Afterward, collect the mixed rumen fluid and immediately transfer it to the laboratory, storing it at 39 °C as a backup. The culture medium was used in this study as follows (Table 1), and all the medium was autoclaved (121 °C, 15 min). It is worth noting that the medium was supplemented with FG after autoclaving.

**Table 1:** Preparation of medium components

No.	Medium components
1.	S-Screening medium ^1^
2.	L-LB, S-LB ^2^
3.	L-MRS, S-MRS ^3^
4.	TGFG L-MRS^4^
5.	GFFG L-MRS ^5^
6.	HGFG L-MRS ^6^

^1^ S-Screening medium: the solid media with 0.5% (w/v) (NH_4_)_2_SO_4_, 0.1% (w/v) KH_2_PO_4_, 0.1% (w/v) NaCl, 0.05% (w/v) MgSO_4_.7H_2_O, 0.01% (w/v) CaCl_2_, 0.02% (w/v) yeast extract, 1.5% (w/v) agar and 0.1% (w/v) free gossypol as the sole carbon source;

^2^ L-LB: the liquid media prepared from the composition of standard LB broth (China Haibo Biotechnology Co., Ltd., Qingdao, China). S-LB: the solid media with L-LB medium and 1.5% (w/v) agar;

^3^ L-MRS: the liquid media prepared from the composition of standard Man-Rogosa-Sharpe (MRS) broth (China Haibo Biotechnology Co., Ltd., Qingdao, China). S-MRS: the solid media with L-MRS medium and 1.5% (w/v) agar;

^4^ TGFG L-MRS: the L-MRS medium with 0.01% (w/v) free gossypol;

^5^ GFFG L-MRS: the L-MRS medium with glucose-free but 0.01% (w/v) free gossypol;

^6^ HGFG L-MRS: the L-MRS medium with half glucose (1% (w/v) glucose) and 0.01% (w/v) free gossypol;

### Isolation and purification of bacteria

All subsequent experimental steps were carried out in an anaerobic environment by YQX-II anaerobic incubator (Shanghai Haixiang Instrument Equipment Factory., Shanghai, China). The 5 mL rumen fluid sample was decimally diluted (10^− 1^, 10^− 2^) with the 39 °C saline solution, then the dilution solution was streaked with a sterilized wire loop onto the S-Screening medium plates [16] for 72 h in the AY6907 anaerobic jar (GeneScience, Wilmington, USA). The bacteria in a single colony arbitrarily selected from colonies on the S-Screening medium (solid medium with FG as the sole carbon source, shown in Table 1) plate were inoculated into L-LB medium (liquid medium based on standard LB broth) and cultured for 24 h. Subsequently, the enrichment cultures were decimally diluted (10^− 2^) with the 39 °C saline solution, and streaked onto the S-Screening medium plate for 72 h again. The process was reiterated until a consistent isolation of the purified single colony morphology was achieved, identifying it as the target strain [17]. The pure isolates were stored at -80 °C in 50% glycerol containing Man-Rogosa-Sharpe (MRS) medium until use.

### Characterization and identification of the isolated bacteria

As the initial characterization of isolated bacteria, a small portion of the culture suspension was spotted on a sterile glass slide and stained by a bacterial Gram-staining kit, according to the manufacturer′s instruction [18]. The DNA was extracted using the Ezup Column Bacteria Genomic DNA Purification Kit (Sangon Biotech Co., Ltd, Shanghai, China). The DNA concentration and purity were checked by a Nanodrop 2000 (Thermo Scientific, Wilmington, USA), and then used as a template for polymerase chain reaction (PCR) amplification. The universal primer sequences were 27 F: 5′-AGAGTTTGATCCTGGCTCAG-3′ and 1492R: 5′-ACGGTTACCTTGTTACGACTT-3′, and the PCR reaction was conducted using the following conditions: The reaction procedure was pre-denaturation at 94 °C for 5 min; 94 °C denaturation for 30 s; annealing at 55 °C for 30 s; and 72 °C extension for 90 s. This was performed for 35 cycles. Finally, amplification was completed by incubation for 5 min at 72 °C [19]. The amplified products were sent to Sangon Biotech Co., Ltd, Shanghai, China. The nucleotide sequences were analyzed for sequence identity by BLAST in the GenBank of NCBI (https://blast.ncbi.nlm.nih.gov/Blast.cgi). To obtain the GenBank accession numbers, the 16 S rRNA sequences of strains were uploaded into NCBI databases. The neighbor-joining method (1000 bootstrap value) of MEGA 6.0 software was used to construct a phylogenetic tree to visualize the phylogenetic relationships [20].

### Determination of the growth curve of the target strain

Activated culture: the stored strain in glycerol at -80 °C was thawed and cultivated in S-MRS medium (solid medium based on standard MRS broth), followed by subculturing twice in L-MRS medium (liquid medium based on standard MRS broth). The 2 mL of activated culture was inoculated onto 200 mL L-MRS medium and cultured at 39 °C for 24 h (12 incubation time × 3 replicates). Every 2 h, the colony forming units in CFU/ml (determined after incubation by plating 100 µL bacteria solution onto S-MRS plates at 39 °C for 24 h), the OD600 value (determined by the UV-2000 model UV/visible spectrophotometer (UNIAC Instrument Co., Ltd., Shanghai, China)), and pH values (measured by the PHS-2 F model pH meter (OUSTOR Industrial Co., Ltd., Shanghai, China)) were obtained. Plate colony counts and plate photographs were taken using the Interscience Scan 4000 (France Interscience Co., Ltd., Cantal, France).

### Detoxification for free gossypol and solid-state fermentation of cottonseed meal

The detoxification experiments were conducted with different free gossypol levels and carbon sources. Activated culture, l mL saline solution and 1 mL culture suspensions (around to 10^8^ CFU/mL) were inoculated onto the 100 mL following fresh medium labeled, respectively: CONL (saline solution + L-MRS medium (2% (w/v) glucose) with 0.01% (w/v) free gossypol), GFFG (culture suspensions + L-MRS medium with glucose-free but 0.01% (w/v) free gossypol), HGFG (culture suspensions + L-MRS medium with half glucose (1% (w/v) glucose) and 0.01% (w/v) free gossypol), TGFG (culture suspensions + L-MRS medium (2% (w/v) glucose) with 0.01% (w/v) free gossypol), and incubated for 24 h at 39 °C. The bacterial solution samples were taken at 0, 6, 12, and 24 h to obtain the degradation efficiency of FG (D_FG_) and OD600 value, and calculated from the formula as follows:

$${\% D}_{FG}=({C}_{0}-{C}_{T})/{C}_{0}*100$$

Where *C*_*T*_ is the FG concentration in the medium at time t = 6, 12, 24 h, and *C*_*0*_ is the FG concentration in the medium at time t = 0. The FG concentration was quantified by High Performance Liquid Chromatography (HPLC) with a Wufeng analytical instrument (Wufeng Co., Ltd., Shanghai, China) as the methods of Wang et al. [21].

To practically evaluate the degradation ability of FG in cottonseed meal (CSM) and fermentation performance of CSM for *Lact. mucosae* LLK-XR1, the experiment of solid-state fermentation from CSM was organized. The procured substrates of CSM were not ground to powder, it was performed with the ratio of material to water of 1:1 and the substrates were inoculated with 8% culture suspensions (around 10^8^ CFU/mL). The trials were grouped into 4 blocks of 3 replicates, each labeled as CON (did not any treatment); SAF (saline solution + autoclaved substrates); CAF (culture suspensions + autoclaved substrates); CUF (culture suspensions + unautoclaved substrates). Then they were incubated for 5 d at 39 °C. After freeze-drying and crushing, the substrate samples were taken at D 3 and D 5 to obtain D_FG_.

### Carbohydrate metabolism

Meanwhile, to clarify any uncertain enzymatic activity found in the selected potential probiotic *Lact. mucosae* LLK-XR 1, the API 50 CHL kit (BioMerieux, Marcy I’ Étoile, France) was used in this study, according to the manufacturer’s instructions [22]. This method uses the color change characteristics of the bromocresol violet indicator when exposed to acid and evaluates the metabolic ability of specific substrates.

### Assessment of temperature tolerance

Activated culture, 10 mL activated cultures (10^8^ CFU/mL) was centrifuged to collect stationary-phase cells (10,000 × g for 10 min at 4 °C), washed twice using sterile PBS (Beijing Solarbio Science & Technology Co., Ltd., Beijing, China) and resuspended in PBS to initial volume. The bacteria suspension in 15 mL tubes was incubated for 120 min at 15, 25, 55, 39, and 65 °C. Suspension samples were collected at 0 and 120 min, followed by serial dilutions were prepared and plated onto S-MRS plates to obtain colony forming units. Tests were performed independently in triplicates to determine the Resistance index (RI), and calculated from the formula as follows [23].

$$\% RI = (\log {\text{ }}CFU{\text{ }}at{\text{ }}time = t/\log {\text{ }}CFU{\text{ }}at{\text{ }}time = o)*100$$

### Assessment of low pH and bile tolerance

Activated culture, 1 mL activated cultures were incubated in 15 mL tubes of 9 mL modified L-MRS for 120 min at 39 °C according to [24]. The modified L-MRS medium was adjusted to pH 2.0, 3.0, 4.0, 5.0, 6.0, or supplemented with 0.2%, 0.3%, 0.4%, 0.5%, 0.6% (w/v) bovine bile salt (Beijing Solarbio Science & Technology Co., Ltd., Beijing, China), the L-MRS medium at pH 6.7 or without bovine bile salt was served as a control. The steps to collect samples (only at 0 and 120 min) and obtain colony forming units, and calculated from the formula as follows:

$$\% RI = (\log {\text{ }}CFU{\text{ }}at{\text{ }}time = t/\log {\text{ }}CFU{\text{ }}at{\text{ }}time = o)*100$$

### Assessment of antibiotic resistance

Antibiotic resistance of *Lact. mucosae* LLK-XR1 to the used antibiotics was evaluated by the Kirby-Bauer disc diffusion method. The following antibiotic discs (BKMAM Biotechnology Co., Ltd, Hunan, China) included: penicillin (10 µg), ampicillin (10 µg), ceftriaxone (30 µg), tetracycline (30 µg), erythromycin (15 µg), ciprofloxacin (5 µg), lincomycin (2 µg), compound sulfamethoxa (25 µg), chloramphenicol (30 µg), gentamycin (10 µg), amikacin (30 µg). Activated culture, the activated cultures with 0.5 McFarland turbid were homogeneously swabbed into S-MRS plates, followed by each of three antibiotic discs being dispensed on a plate. After anaerobic incubation for 48 h at 39 °C, inhibitory zones were recorded using by Interscience Scan 4000, and results were reflected as sensitive or resistant based on CSLI 2012 guidelines [25].

### Assessment of antimicrobial activities

The most common pathogenic microbes used in this study, including *Escherichia coli* CICC 10,899, *Staph. aureus* CICC 21,600, and *Salmonella. typhimurium* CICC 21,483, were provided by the China Center of Industrial Culture Collection. Antipathogenic activities of cell-free supernatants of *Lact. mucosae* LLK-XR1 were assessed carried out as described by Jabbari et al. [26]. Activated culture, the activated cultures (10^8^ CFU/mL) were centrifuged to collect the supernatant (10,000 × g for 10 min at 4 °C), then sterilized to remove stationary-phase cells using 0.22 μm cellulose acetate membrane filter (Beijing Solarbio Science & Technology Co., Ltd., Beijing, China). Suspension of pathogenic bacteria cultured in L-LB was swabbed into S-LB medium (Solid medium based on standard LB broth), then plates (3 pathogenic bacteria × 3 replicates) were injected with 80 µL of cell-free supernatants into an 8 mm well, while the equal volume of sterile saline plate was served as the control. Following incubation for 24 h at 37 °C, plates were observed for the inhibitory zone. Referring to the description of a previous study, the inhibitory zone diameter is 7–9 mm indicated weak antimicrobial activities, 10–13 mm indicated intermediate antimicrobial activities, 14–17 mm indicated strong antimicrobial activities, and > 17 mm indicated very strong antimicrobial activities [26].

### Assessment of cell surface properties

Assessment of cell surface properties was performed according to the procedure described by Muñoz-Provencio et al. [27]. Activated culture, 10 mL activated cultures (10^8^ CFU/mL) were centrifuged (10,000 × g for 10 min at 4 °C), washed twice using sterile PBS (Phosphate buffer saline) and resuspended in PBS to initial volume as the final absorbance (A_0_) at 600 nm. Whereafter, 3 mL bacteria suspension was mixed with 1 ml of organic solvents: hexadecane, chloroform and ethyl acetate, respectively. The mixture was thoroughly vortexed for 1 min and incubation at 39 °C for 30 min, then absorbance (A_1_) at 600 nm of the aqueous layer was determined. *E. coli* CICC 10,899 and *Salm. Typhimurium* CICC 21,483 were used as the control. The percentage of affinity (% PA) was calculated from the formula as follows:

$$\% PA=({A}_{0}-{A}_{1})/{A}_{0}*100$$

### Assessment of auto-aggregation ability

*Assessment of a*uto-aggregation was performed as described by Kos et al. [28]. Activated culture, the pre-treatment was conducted for obtaining bacterial suspension consistent with the previous description. The bacterial suspension was thoroughly vortexed for 1 min, and recorded the absorbance (A_0_) at 600 nm. Follow by incubation at 39 °C for 24 h, suspension samples were taken at different times (at 2, 6, 12, 24 h) to determine the absorbance (A_t_). Percentage of auto-aggregation (% AA) was calculated from the formula as:

$$\% AA=({A}_{0}-{A}_{t})/{A}_{0}*100$$

### Assessment of hemolytic and catalase activity

Activated culture, the cultures were spotted onto blood agar plates (BKMAM Biotechnology Co., Ltd, Hunan, China) containing 5% (v/v) sterile defibrinated sheep blood agar, followed by incubation at 39 °C for 48 h. According to Cui et al. [29], the presence of greenish halos around the colonies indicates α-hemolytic, a clear halo indicates β-hemolytic, and no halo indicates γ-hemolytic (non-hemolytic). *Staph. aureus* CICC 21,600 was used as positive control for β-hemolytic.

The production of intracellular enzyme catalase was also crudely determined [22]. Activated culture, the activated cultures (around to 10^8^ CFU/mL) were spotted on S-MRS plates. Followed by incubation at 39 °C for 24 h, the bacterial colony plates were flooded with 3 mL of 10% (v/v) hydrogen peroxide solution. The presence of gas bubbles on the colonies indicates a positive result.

### Statistical analysis

All the experiments were carried out in triplicates, the experiment data are presented as mean ± standard deviation (SD). Statistical analyses were completed using the general linear model procedure of SAS [30]. The data, including the degradation efficiency of FG, OD 600 of LLK-XR1 under various carbon sources, and microbial adhesion, underwent one-way analysis of variance (ANOVA). The significant differences were analyzed by Duncan’s multiple comparison analysis. The replicate test served as the experimental unit. * *P* value < 0.05 and ** *P* value < 0.01 was considered statistically significant. The growth curve of *Lact. mucosae* LLK-XR1 are performed with Origin version 2021 (OriginLab, Massachusetts USA), and other data are performed with ChiPlot software (https://www.chiplot.online).

## Results

### Initial characterization of the isolated bacteria

A single strain was isolated from the rumen liquid of the healthy Dorper × Hu hybrid sheep with long-term feeding cotton meal by the basal medium plates containing 0.5 g/L gossypol as the sole carbon source. Meanwhile, the isolated Bacteria were identified as Gram-positive bacteria.

### 16 S rDNA sequencing identification and homologous analysis

**Fig. 1:**
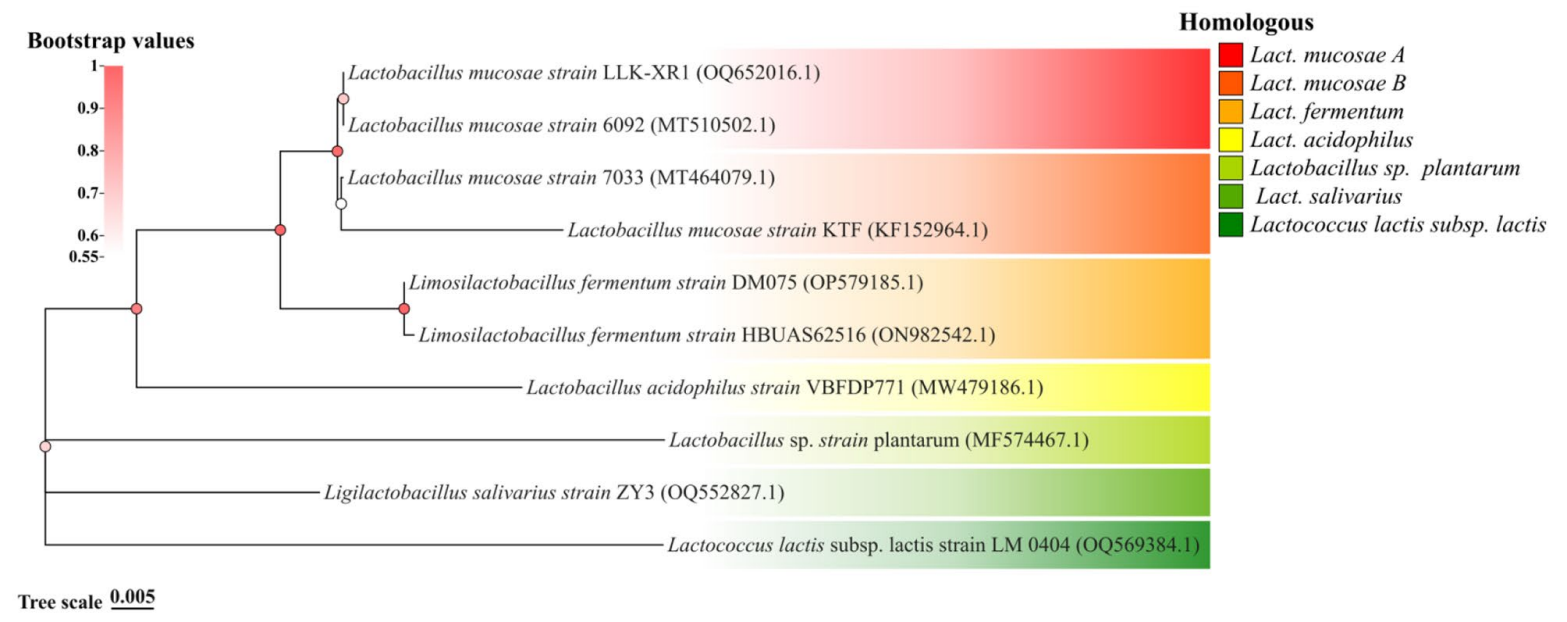
Phylogenetic tree generated using neighbor-joining analysis based on 16 S rRNA gene sequences of *Lact. mucosae* LLK-XR1 and other reported *Lactobacillus* strains. The color of the circle on the branches indicates bootstrap values, and the color of the square indicates homology similarity

The electrophoresis results of the amplified fragments presented the 1466 bp. The 16 S rDNA sequence of the isolated bacteria was submitted to NCBI GenBank database and labeled as LLK-XR1 (accession numbers: OQ652016.1). In the phylogenetic tree (Fig. 1), it indicated that LLK-XR1 and *Lact. mucosae* 6092 are clustered on the same branch. Furthermore, the 16 S rDNA sequences analysis showed that LLK-XR1 was identified as *Lact. mucosae* strain based on the 99.73% homologous with *Lact. mucosae* 6092 in NCBI BLAST.

### Growth curve of *Lact. mucosae* LLK-XR1

**Fig. 2:**
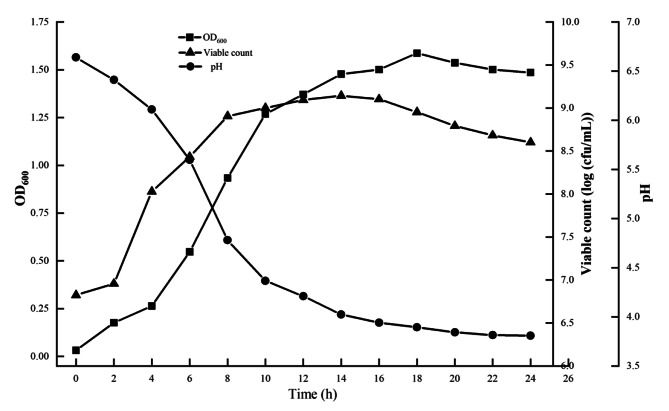
Growth Curve of *Lact. mucosae* LLK-XR1. The values were means with three technical replicates

The lag growth phase of *Lact. mucosae* LLK-XR1 was observed over the first 2 h of anaerobic culture at 39 °C (Fig. 2). Then, it arrived in the logarithmic growth phase during the period of 2 to 8 h, while reached the plateau phase at 8 h, with viable count at 8 and 14 h of 8.906 log (CFU/mL) and 9.143 log (CFU/mL). The results of OD_600_ were generally consistent with the results of viable count in reflecting the bacterial growth stage. The pH values of the medium dropped from 6.64 to 3.81 during the entire incubation period.

### Growth of *Lact. mucosae* LLK-XR1 and detoxification of FG under different carbon source

The growth of *Lact. mucosae* LLK-XR1 and its D_FG_ under different carbon sources were illustrated in Table 2. A significant (*P* < 0.01) increase in OD600 value of Group HGFG and Group TGFG was observed at 6 and 12 h compared with Group GFFG when treated with the glucose concentration in the medium. At 24 h, the results showed the same increase (*P* < 0.01) in OD600 value of decline phase of *Lact. mucosae* LLK-XR1 with increasing glucose concentration among all treatments. Meanwhile, when stratifying the intra-group differences by time, the significant (*P* < 0.01) increase of OD600 value for Groups GFFG and TGFG, but significantly (*P* < 0.01) higher levels of OD600 value were observed at 12 h than at 24 for Group HGFG.

**Table 2:** Effects of different carbon sources on the growth of *Lact. mucosae* LLK-XR1 and its degradation rate of free gossypol

Items	Treatments				SEM	*P*-value
	CONL	GFFG	HGFG	TGFG		
OD600						
6 h	/	0.072 ^aA^	0.678 ^bA^	0.682 ^bA^	0.022	< 0.01
12 h	/	0.165 ^aB^	1.171 ^bB^	1.219 ^bB^	0.017	< 0.01
24 h	/	0.451 ^aC^	0.890 ^bC^	1.334 ^cC^	0.010	< 0.01
D_FG_ (%)						
6 h	1.261 ^a^	31.387 ^bA^	42.836 ^cA^	6.581 ^dA^	0.769	< 0.01
12 h	1.751 ^a^	47.354 ^bB^	59.863 ^cB^	46.305 ^bB^	1.525	< 0.01
24 h	1.443 ^a^	47.644 ^bB^	59.926 ^cB^	69.512 ^dC^	0.962	< 0.01

CONL: saline solution & L-MRS medium with 0.01% (w/v) free gossypol; GFFG: culture suspensions + L-MRS medium with glucose-free but 0.01% (w/v) free gossypol; HGFG: culture suspensions+ L-MRS medium with half glucose (1% (w/v) glucose) and 0.01% (w/v) free gossypol; WGFG: culture suspensions + L-MRS medium with 0.01% (w/v) free gossypol;

^a, b, c^ Values in a line within the same class without a common superscript are significantly different (*P* < 0.05);

^A, B, C^ Values in a column within the same class without a common superscript are significantly different (*P* < 0.05)

The results indicated that there were significant (*P* < 0.01) differences in the D_FG_ among all treatments at 6 and 24 h, but the specific between-group differences showed inconsistent. At the 6 h, results showed that the D_FG_ was highest in Group HGFG (42.836%), then in Group GFFG (31.387%), and lowest in Group TGFG (6.581%). However, Group TGFG had the highest levels of the D_FG_ (69.512%), Group HGFG was the second highest (59.926%), and Group GFFG was the lowest (47.644%) at 24 h. At the 12 h, significantly (*P* < 0.01) higher levels of the D_FG_ of FG were observed in Group HGFG (59.863%) than in Groups GFFG (47.354%) and TGFG (46.305%), without significant differences (*P* > 0.05) between Groups GFFG and TGFG. When stratifying the intra-group differences by time, significantly (*P* < 0.01) lower levels of the D_FG_ were observed at 6 h than at 12 and 24 h for Groups GFFG and HGFG, and a significant increase (*P* < 0.01) in the D_FG_ over time was observed for Group TGFG. Overall, Group differences were observed in the D_FG_ for Group TGFG performing worse initially, but better toward the end.

### Detoxification of FG using solid-state fermentation of cottonseed meal by *Lact. mucosae* LLK-XR1

**Fig. 3:**
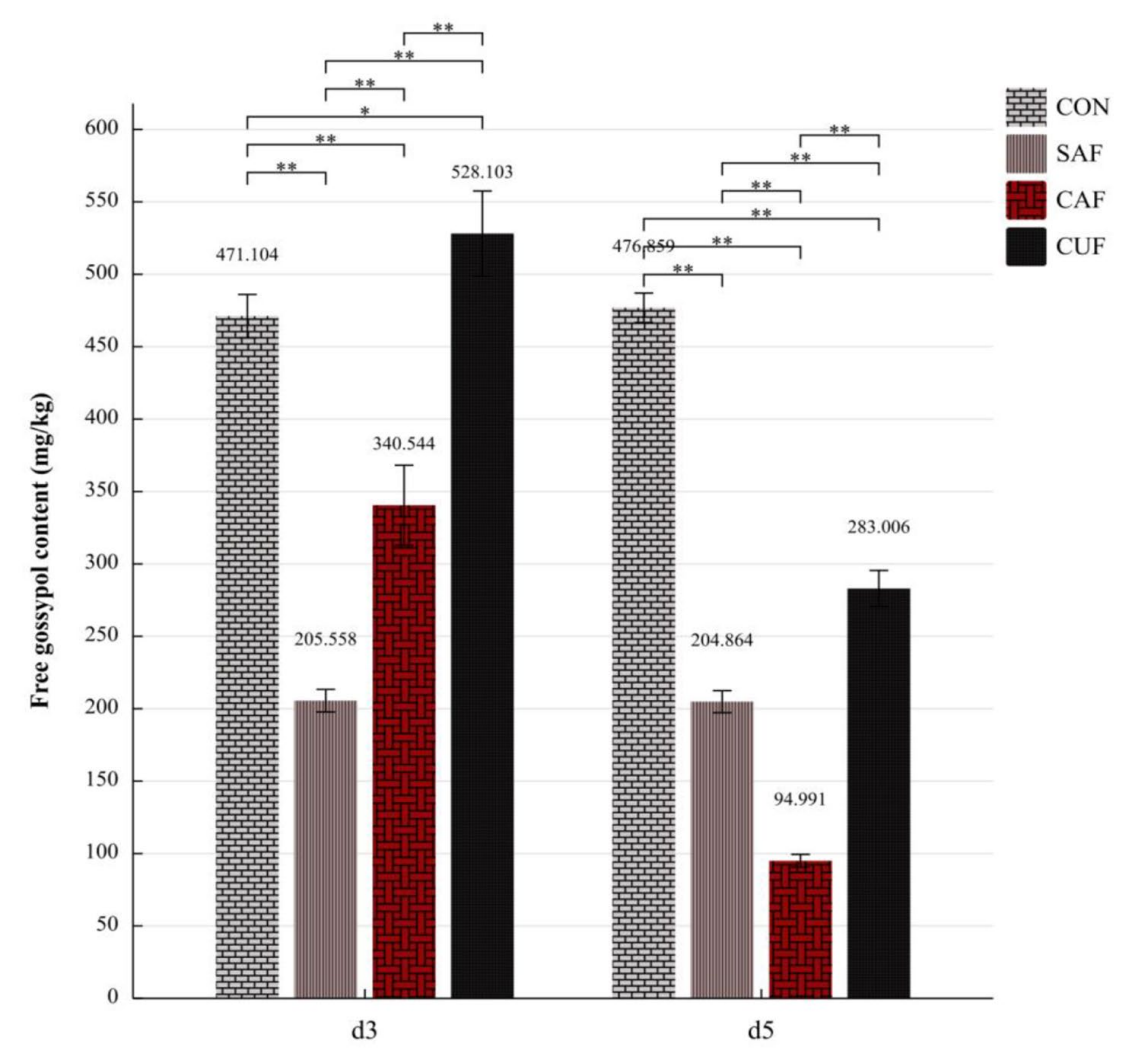
The change of free gossypol concentrations in solid-state fermentation of cottonseed meal by *Lact. mucosae* LLK-XR1. CON: did not any treatment; SAF: saline solution + autoclaved substrates; CAF: culture suspensions + autoclaved substrates; CUF: culture suspensions + unautoclaved substrates. The mean values with three technical replicates are expressed above the bars, and the error bars represent standard deviations. The “ * ” and “ ** ” indicate the significant difference (*P* < 0.05) and extremely significant difference (*P* < 0.01) between bars, respectively

The change of FG content using solid-state fermentation of CSM under different treatments was shown in Fig. 3. The results indicated that there were significant (*P* < 0.05) in the FG content at d 3 and d 5 among all treatments. The specific performance was that the FG content was highest in Group CUF (528.103 mg/kg), followed by Group CON (471.104 mg/kg), then in Group CAF (340.544 mg/kg), and lowest in Group SAF (205.558 mg/kg) at d 3. The mean value of FG content did not change significantly (*P* > 0.05) over time covering Groups CON and SAF. After d 5 of fermentation, FG content was highest in Group CON (476.859 mg/kg), followed by Group CUF (283.006 mg/kg), then in Group SAF (204.864 mg/kg), and lowest in Group CAF (94.991 mg/kg). The 80.08% lower in Group CAF of FG content compare to Group CON (*P* < 0.01), and 40.652% lower in Group CUF of FG content than Group CON (*P* < 0.01).

### Carbohydrate metabolism profile

After the incubation by API 50 CHL kit, *Lact. mucosae* LLK-XR1 demonstrated color changes for metabolizing various carbohydrates (Table 3). The specific performance is that the LLK-XR1 strain positive for L-Arabinose, Ribose, D-Xylose, β-Methyl Xylose, Galactose, D-Glucose, D-Fructose, D-Manose, Amygdaline, Maltose, Lactose, Melibiose, Saccharose, Melizitose, D-Raffinose, Gluconate, 2-ceto-gluconate, 5-ceto-gluconate.

**Table 3:** Utilized Carbohydrates by *Lact. mucosae* LLK-XR1 by using API 50 CHL Kit

Type of carbohydrate source	Results	Type of carbohydrate source	Results
Glycerol	-	Esculine	-
Erythritol	-	Salicine	-
D-Arabinose	-	Cellobiose	-
L-Arabinose	+	Maltose	+
Ribose	+	Lactose	+
D-Xylose	+	Melibiose	+
L-Xylose	-	Saccharose	+
Adonitol	-	Trehalose	-
β-Methyl Xylose	+	Insuline	-
Galactose	+	Melizitose	+
D-Glucose	+	D-Raffinose	+
D-Fructose	+	Amidon	-
D-Manose	+	Glycogene	-
L-Sorbose	-	Xylitol	-
Rhamnose	-	β-Gentibiose	-
Dulcitol	-	D-Turanose	-
Inositol	-	D-Lyxose	-
Mannitol	-	D-Tagalose	-
Sorbitol	-	D-Fucose	-
β-Methyl-D-mannoside	-	L-Fucose	-
β-Methyl-D-glucoside	-	D-Arabitol	-
N-acetyl glucosamine	-	Gluconate	+
Amygdaline	+	2-ceto-gluconate	+
Arbutine	-	5-ceto-gluconate	+

“+” indicates utilized; “-” indicates not utilized

### Temperature, low acid, and bile tolerance

*Lact. mucosae* LLK-XR1 exhibited varying degrees of reduction (Table 4) in the final population in terms of log CFU/mL after 120 min exposure to other temperatures or pH as compared to 39 °C (97.68%) or pH 6.0 (98.30%). At 15 and 65 °C, LLK-XR1 showed 63.53 and 12.07% mean RI, respectively. Whereas at pH 2.0 and 3.0, LLK-XR1 displayed 37.92 and 88.63% mean RI. LLK-XR1 can appreciably tolerate 0.2% bile concentration for 80.64% RI, However, in this study, when LLK-XR1 was exposed to above 0.5% bile salt concentrations for 120 min, no cells survived.

**Table 4:** In vitro resistance to temperature, acid, and bile stress of *Lact. mucosae* LLK-XR1

Items	RI (%)		RI (%)		RI (%)
Temperature (°C)		pH		Bile salt (%)	
15	63.53 ± 4.67	2.0	37.92 ± 3.42	0.2	80.64 ± 3.47
25	90.40 ± 3.04	3.0	88.63 ± 1.90	0.3	78.52 ± 2.06
39	97.68 ± 0.88	4.0	92.57 ± 2.01	0.4	43.10 ± 6.39
55	38.61 ± 3.30	5.0	96.14 ± 2.71	0.5	0
65	12.07 ± 1.58	6.0	98.30 ± 1.25	0.6	0

“RI” indicates the resistance index to the stress

### Antibiotic susceptibility

To ensure the safe utilization of *Lact. mucosae* LLK-XR1, it is recommended to conduct the assessment of their antibiotic susceptibility profile. In this study, the zones of clearance were observed against 11 antibiotics present in Table 5. LLK-XR1 was sensitive to Erythromycin, Ampicillin, Amikacin, Chloramphenicol, Ceftriaxone, Lincomycin, Gentamycinm Tetracycline, Penicillin with a variable and significant zone of inhibition. LLK-XR1 was resistant to Compound Sulfamethoxazole, Ciprofloxacin and Gentamycin.

**Table 5:** Antibiotic susceptibility profile of *Lact. mucosae* LLK-XR1

Antibiotics	Disc potency (ug)	Inhibitory zones (mm)	Antibiotic Susceptibility
Erythromycin	15	29.13 ± 0.15	S
Ampicillin	10	17.54 ± 0.23	I
Amikacin	30	15.33 ± 0.32	I
Chloramphenicol	30	28.033 ± 0.15	S
Compound Sulfamethoxazole	25	negative	R
Ciprofloxacin	5	negative	R
Ceftriaxone	30	32.23 ± 0.15	S
Lincomycin	2	27.17 ± 0.21	S
Gentamycin	10	negative	R
Tetracycline	30	26.70 ± 0.17	S
Penicillin	10	25.63 ± 0.16	S

“R” indicates resistance to antibiotics; “I” is intermediary susceptible to antibiotics; S indicates susceptible. Inhibition halo interpreted according to CLSI 2012 guidelines

### Antimicrobial activities

The ability of *Lact. mucosae* LLK-XR1 to exhibit antimicrobial activities against potent enteric pathogens further highlights its significant role as the potential probiotic in preventing infections caused by pathogenic microbes within the gastrointestinal tract. The cell-free supernatants of *Lact. mucosae* LLK-XR1 showed varying inhibition zones against three distinct enteric pathogens used in the study (Table 6). *Lact. mucosae* LLK-XR1 exhibited strong inhibition against *E. coli* CICC 10,899 and *Staph. aureus* CICC 21,600, with inhibition zone sizes of 14 to 17 mm. In contrast, *Salmonella. Typhimurium* CICC 21,483 was intermediately inhibited, with inhibition zone sizes ranging from 10 to 13 mm.

**Table 6:** Antimicrobial activities of *Lact. mucosae* LLK-XR1

Indicator strain	Antimicrobial activities
*E. coli* CICC 10,899	★★★
*Staph. aureus* CICC 21,600	★★★
*Salmonella. Typhimurium* CICC 21,483	★★

The antimicrobial activities of cell-free culture supernatant of *Lact. mucosae* LLK-XR1 was indicated as no inhibition, ★weak inhibition (7–9 mm), ★★intermediate inhibition (10–13 mm), ★★★ strong inhibition (14–17 mm) and ★★★★very strong inhibition (> 17 mm)

### Cell surface properties

**Fig. 4:**
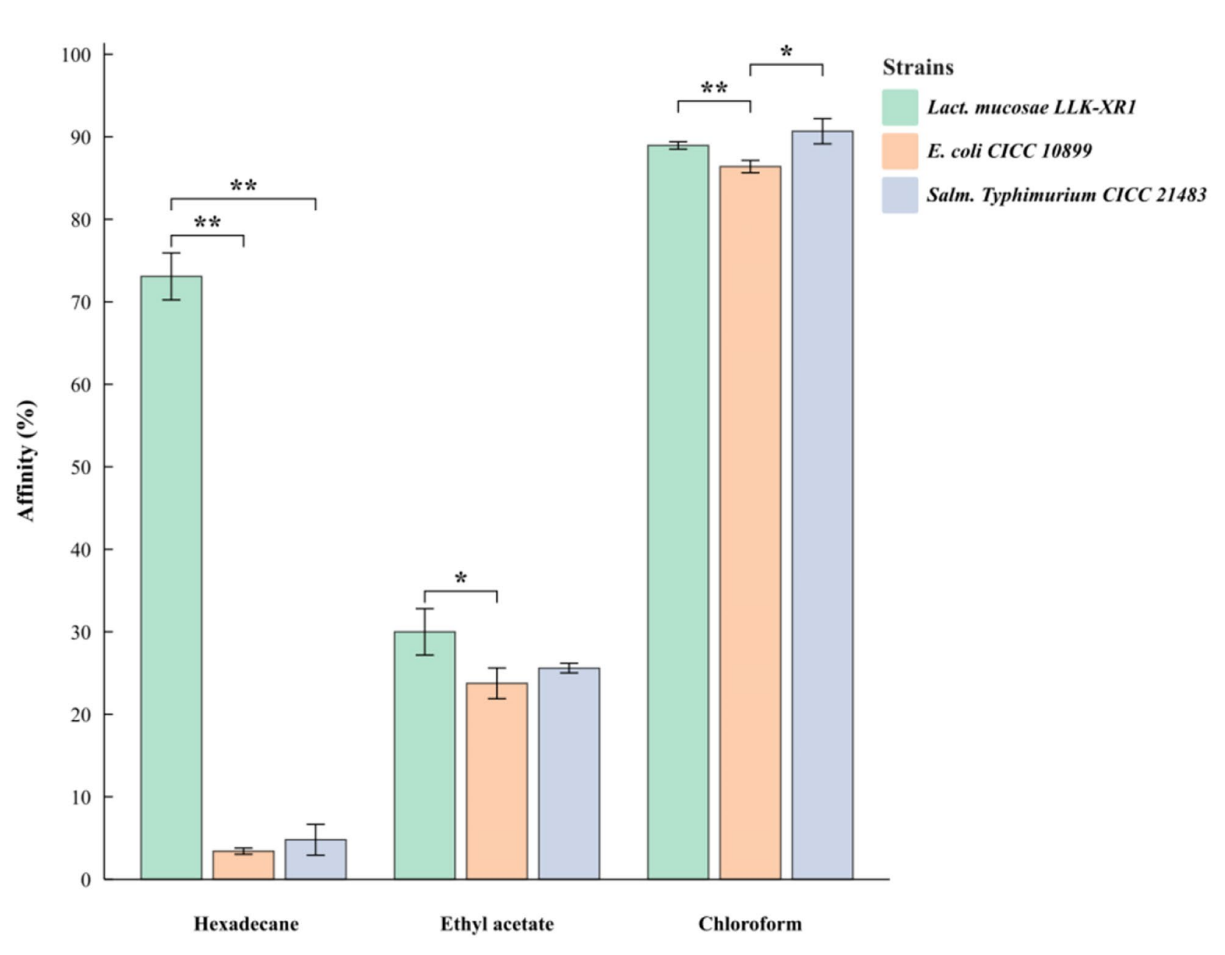
Microbial adhesion to solvents test of *Lact. mucosae* LLK-XR1. *E. coli CICC* 10,899 and *Salm. Typhimurium* CICC 21,483 were used as the control. The values were means with three technical replicates, and error bars represent standard deviations. The “ * ” and “ ** ” indicate the significant difference (*P* < 0.05) and extremely significant difference (*P* < 0.01) between bars, respectively

The ability of bacterial adhesion of *Lact. mucosae* LLK-XR1 to adhere to the digestive tract epithelial cells was evaluated based on its cell surface hydrophobicity towards hexadecane, ethyl acetate and chloroform. *Lact. mucosae* LLK-XR1 showed 73.079% affinity to hexadecane, which was significantly (*P* < 0.01, Fig. 4) higher than *E. coli* CICC 10,899 (3.410%) and *Salmonella. Typhimurium* CICC 21,483 (4.797%). While *Lact. mucosae* LLK-XR1 showed 29.995% affinity to ethyl acetate, which was significantly (*P* < 0.05) higher than *E. coli* CICC 10,899 (23.757%), no significant (*P* > 0.05) difference was observed in other between-group. The results indicated that the affinity of *Lact. mucosae* LLK-XR1 determined with chloroform (> 88.951%) was significantly (*P* < 0.01) higher than *E. coli* CICC 10,899 (86.39%), and no significant (*P* > 0.05) difference of the affinity between *Lact. mucosae* LLK-XR1 and *Salmonella. Typhimurium* CICC 21,483 (90.676%) to chloroform.

### Auto-aggregation capacity

**Fig. 5:**
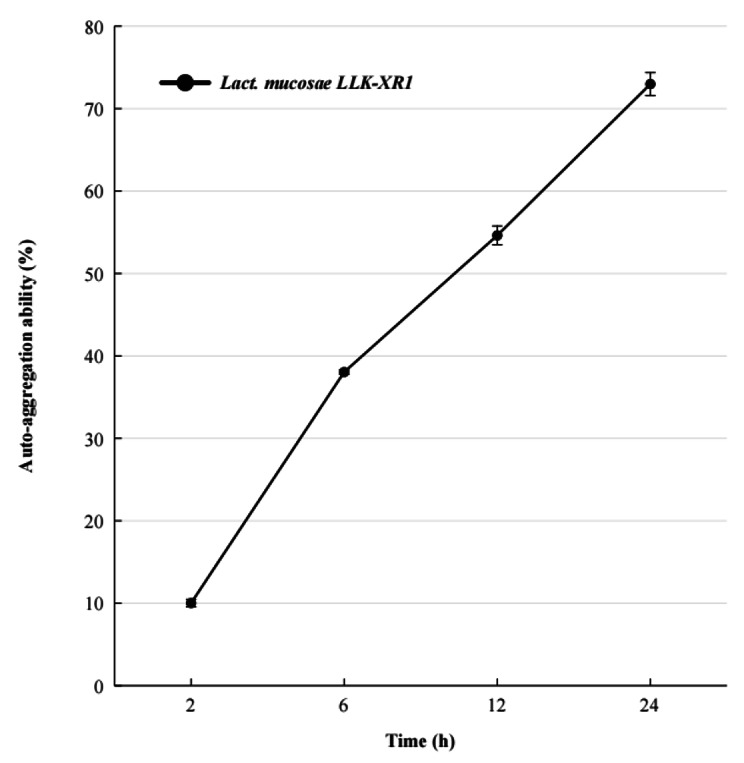
Cellular auto-aggregation ability of *Lact. mucosae* LLK-XR1. The values were means with three technical replicates, and error bars represent standard deviations

Cellular auto-aggregation ability of *Lact. mucosae* LLK-XR1 was measured at 2 h, 6 h, 12 h, and 24 h, and results are expressed in Fig. 5, which showed a continuous augmentation in auto-aggregation by *Lact. mucosae* LLK-XR1. It showed the fastest increase rate (AA/h) in auto-aggregation from 2 h (10.016%) to 6 h (38.044%), followed by from 6 to 12 h (54.62%), lowest from 12 to 24 h (72.981%). While the highest ability to auto-aggregate of *Lact. mucosae* LLK-XR1 after 24 h.

### Hemolytic and catalase activity

To determine the suitability of *Lact. mucosae* LLK-XR1 as potential probiotics, their safety properties were evaluated. Specifically, in our study, *Lact. mucosae* LLK-XR1 exhibited γ-hemolytic (non-hemolytic) when cultured on 5% (v/v) sterile defidrinated sheep blood agar as compared to control *Staph. aureus* CICC 21,600.

## Discussion

The previous studies have indicated the significant capability of the rumen microorganisms in gossypol biodegradation [3, 6, 9]. In the current study, target strain was isolated from rumen liquid by the screening medium with free gossypol as the sole carbon source. However, there’s a potential concern here: rumen microbes that can utilize other carbon sources may also have the capability to degrade free gossypol. These microbes might not be isolated using this screening method. Other researchers may choose to conduct experiments by purifying first and then validating the degradation of free gossypol according to the actual situation. This study’s screening approach was designed to precisely isolate the desired strains. Then we identified LLK-XR1 according to morphological and molecular methods [17–20], and evaluated the growth curve, which is consistent with previous studies on *Lactobacillus* strains [31, 32]. Meanwhile, LLK-XR1 exhibited efficiency for degrading FG (47.644%) at 24 h when used as the sole carbon source in a liquid medium. Wang et al. [16] isolated *Lact. agilis* strain from the rumen with high activity of D_FG_ (49.25%), which is slightly higher than LLK-XR1. The different FG concentrations in the liquid medium (0.1 g/L in this study vs. 1 g/L in Wang’s study [16]) and the different strains could be a contributing factor to this observation. Therefore, it can be stated that LLK-XR1 can utilize FG as a carbon source from this part of the results, but further investigation is needed to precisely quantify its degradation efficiency. This is the first reported instance in the present study of a *Lact. mucosae* strain isolated from the rumen exhibiting the ability to degrade FG. In addition, in a liquid medium with equal concentrations of FG, Group TGFG exhibited the lowest D_FG_ but had higher OD600 values at 6 h, suggesting the competitive relationship between glucose and FG as carbon sources for LLK-XR1 utilization. And, it is evident that glucose was more dominant in this competition. The higher glucose concentration in the medium at 24 h, the higher the D_FG_ by LLK-XR1, suggesting that modifying the nutrient levels in the fermentation medium could tag the potential to enhance FG degradation of LLK-XR1. While the OD600 values of LLK-XR1 in Group TGFG (the L-MRS medium with 0.01% FG in Table 2) were lower than (1.334 at 24 h vs. 1.486 at 24 h) that of Results in Fig. 4 in all time periods, this showed that the FG has a negative effect on the normal growth of LLK-XR1, but LLK-XR1 had a certain tolerance to FG injury.

In recent years, solid-state fermentation has gained attention due to its advantages over submerged fermentation, it offers the potential to utilize inexpensive substrates and obtain value-added feed and food products [33]. In the current study, the D_FG_ by solid-state fermentation using CSM and LLK-XR1 was analyzed. The significant reduction in FG content observed in Group SAF compared to Group CON indicated that high-temperature heat treatment is effective in decreasing the FG contents in CSM. The results obtained in this study are consistent with the previous studies [2, 34]. However, it is noteworthy that the FG content in Group CAF was found to be higher than that in Group SAF, as same as Group CUF compared to Group CON for 3 d of fermentation, which was different from the previous studies [11, 15, 16]. The only difference between these two treatments of two groups comparisons was the addition or not of the culture suspensions of LLK-XR1. After ensuring the accuracy of this study operation, the reasons for the FG content increase may be as follows. To be more relevant to the actual fermentation production procedures, uncrushed CSM was employed as the fermentation substrate in this experiment different compared to the crushed CSM in Wang’s study [16]. For the uncrushed CSM, it is possible that LLK-XR1 initially promotes cellulose decomposition in CSM during the pre-fermentation period [35], resulting in the release of FG and subsequently increasing their content, while the greater proportion of FG may be in a directly exposed state for the crushed CSM. Meanwhile, the LLK-XR1 may release FG from the BG form (produced via covalent bonds between FG and the free epsilon-amino groups from lysine and arginine [1]) according to the results of an increase of lysine [16], or both lysine and arginine after the fermentation [11]. Further investigation is necessary to accurately determine the factors contributing to these discrepancies in the degradation of FG during fermentation. After 5 d fermentation, both Groups CAF and CUF exhibited a reduction in FG content compared to the results of the 3 d fermentation, and lower than Group CON. Despite the lower D_FG_ observed in solid-state fermentation compared to previous studies, it can be attributed to the use of additional nutrients, MRS medium as the diluent for inoculum preparation [16], carbohydrate sources, urea and minerals [15]. Which enhances the viability and abundance of bacteria, thereby improving the D_FG_. Additionally, the use of autoclaved substrates may also contribute to the higher D_FG_ [11, 16]. Overall, LLK-XR1 exhibited a high capacity for the efficient degradation of FG. Nevertheless, based on the results observed in the liquid medium, there is a possibility that its maximum degradation potential remains untapped. Further investigation is warranted to explore the parameters of the fermentation of CSM by LLK-XR1 [36].

In this study, to ensure the utilization as a direct feed or silage inoculant, we identified and evaluated the probiotic potential of the *Lact. mucosae* strain. It was able to withstand stress at a pH of 3.0 and with a 0.3% bile concentration for 2 h, which is essential for survival in the gastrointestinal tract [37]. The LLK-XR1 at the pH of 3.0 displayed greater than 88% survivability, and displayed greater than 78% tolerance to 0.3% bile. Which is comparable to the tolerance exhibited by probiotics that have already been approved for use [38]. Safety is a crucial selection criterion for bacterial strains intended for use in the food and feed industry. Therefore, probiotics are approved for use only if they demonstrate broad-spectrum antibiotic susceptibility [39, 40]. The current study indicates that *Lact. mucosae* LLK-XR1 exhibited susceptibility to most antibiotics tested, except for Compound Sulfamethoxazole, Ciprofloxacin and Gentamycin, which is consistent with previous reports on probiotic validation [41, 42]. Aminoglycoside and sulfamethoxazole resistance have been observed in lactic acid bacteria, and it’s been associated with their natural and intrinsic resistance in many reported studies [43]. These resistances are generally considered to be of non-horizontally transmissible nature type or minimal risk [44].

Another compelling health benefit attributed to probiotics is their ability to prevent and reduce infectious diseases in the gastrointestinal tract [45]. Currently, the commonly used probiotics mainly consist of Lactic acid bacteria, specifically *Lactobacillus* and *Saccharomyces* species. These bacteria are capable of producing, organic acids, ethanol, hydrogen peroxide, several enzymes and bacteriocins, which combined inhibit the proliferation and metabolism of pathogenic bacteria, consequently restoring the equilibrium of the gut microbiota [46, 47]. *Lact. mucosae* SRV5 and SRV10 have been previously demonstrated to exhibit varying degrees of inhibitory effects against pathogens [48], which is consistent with these experimental results. The production of lactic acid by *Lact. mucosae* LLK-XR1 may contribute to the inhibition of pathogen growth through the creation of an acidic environment and its potential bactericidal properties. The drop in pH values from 6.64 to 3.81 after 24 h of incubation may be the probable reason for the antimicrobial activities, but whether there are also bacteriocins or other bioactive substances with antibacterial properties still requires further investigation.

The adhesion ability to the gastrointestinal tract is a pivotal criterion in the selection of probiotics, as it enhances their survivability to effectively exert specific functions and positive health effects [49]. Cell surface properties and auto-aggregation assays are widely acknowledged as direct screening techniques used to assess the adhesion capability of probiotic isolates [50]. For assessment of cell surface properties, hexadecane (apolar solvent) was used to evaluate the hydrophobicity of the cell surface, while ethyl acetate (polar basic solvent) and chloroform (polar acidic solvent) were used to explore the electron acceptor and electron donor properties of the bacterial cell surface [27, 51]. In the present study, *Lact. mucosae* LLK-XR1 showed strong hydrophobic surface characteristics from the results of the 73.079% affinity to hexadecane, which is consistent with other studies on *Lact. mucosae strains* [22, 48]. Bacteria with higher hydrophobicity have been observed to possess a stronger binding affinity towards gastrointestinal epithelial cells [49, 51]. Meanwhile, *Lact. mucosae* LLK-XR1 showed weak basic and poor electron acceptor characteristics (29.995% affinity to ethyl acetate), and highly basic characteristics (88.951% affinity to chloroform) [52]. The results in this study of cell surface properties for both pathogenic microorganisms showed hydrophilic and electron donor properties, similar to the previous studies [53]. The exceptional adhesion ability of *Lact. mucosae* LLK-XR1 compared to *E. coli* CICC 10,899 and *Salm. Typhimurium* CICC 21,483 indicates its capacity to compete for host cell binding sites, thereby inhibiting pathogenic microorganisms and contributing to the health benefits [54].

In our study, we examined the correlations between cell surface properties and the auto-aggregation capacity, which is crucial for underlying mechanisms supporting our overarching hypothesis. The correlation between these two variables sheds light on the pivotal role of cell surface properties in bacterial auto-aggregation, a phenomenon with significant implications in various fields, such as medicine and environmental science. Auto-aggregation is another property exhibited by probiotics that enables the cell aggregates formation, and this property also facilitates their adherence to epithelial cells and mucosal surfaces [55]. In the current study, the percentage of auto-aggregation of *Lact. mucosae* LLK-XR1 after 24 h of incubation was greater than 70%, indicating its strong adhesion ability. Meanwhile, *Lact. mucosae* LLK-XR1 exhibited a negative result (non-hemolytic) on 5% (v/v) sterile defibrinated sheep blood agar, which indicates their safety for use [56]. Moreover, the further validation and exploration of the probiotic properties of the target strains will be undertaken in subsequent stages. Our researchers will substantiate their probiotic characteristics and address potential technical challenges during practical application through cellular assays and animal models.

## Conclusions

In summary, the present study highlights the potential of *Lact. mucosae* LLK-XR1, isolated from sheep rumen liquid, as a promising candidate for the bio-degradation of FG and as a probiotic. Briefly, the D_FG_ in L-MRS reached up to 69.512% for 24 h of anaerobic incubation, the D_FG_ during solid-state fermentation of CSM. Further research is needed to determine the optimal fermentation parameters for solid-state fermentation of CSM to reach the maximize the FG degradation potential. Meanwhile, LLK-XR1 demonstrates potential as a probiotic strain capable of tolerating the harsh gastrointestinal environment. Moreover, LLK-XR1 shows good adhesion, auto-aggregation abilities, sensitive to broad-spectrum antibiotics and inhibits enteropahtogenic microganism, implicationg that the *Lact. mucosae* LLK-XR1 could have promising application prospects in direct-feeding to animals that tolerated the stress of FG and microbial fermentation of cotton by-products.

### Electronic supplementary material

Below is the link to the electronic supplementary material.

Supplementary Material 1

## Data Availability

All relevant data are either presented within the manuscript. The study’s raw data and associated analyses may be obtained from the corresponding author upon reasonable request. The 16 S RNA gene sequences of *Lact. mucosae* LLK-XR1 are available through the National Center of Biotechnology Information under accession number: OQ652016.1. (https://www.ncbi.nlm.nih.gov/nucleotide/OQ652016.1).

## Abbreviations

CON: Did not any treatment
BG: Bound gossypol
CAF: Culture suspensions + autoclaved substrates
CFU: Colony forming unit
CONL: Saline solution + L-MRS medium (2% (w/v) glucose) with 0.01% (w/v) free gossypol)
CSM: Cottonseed meal
CUF: Culture suspensions + unautoclaved substrates
DFG: Degradation efficiency of free gossypol
FG: Free gossypol
GFFG: Culture suspensions + L-MRS medium with glucose-free but 0.01% (w/v) free gossypol
HGFG: Culture suspensions + L-MRS medium with half glucose (1% (w/v) glucose) and 0.01% (w/v) free gossypol
HPLC: High Performance Liquid Chromatography
L-LB medium: Liquid medium based on standard LB broth
L-MRS medium: Liquid medium based on standard MRS broth
MRS: Man-Rogosa-Sharpe
PBS: Phosphate buffer saline
PCR: Polymerase chain reaction
SAF: Saline solution + autoclaved substrates
S-LB medium: Solid medium based on standard LB broth
S-MRS medium: Solid medium based on standard MRS broth
S-Screening medium: Solid medium with free gossypol as the sole carbon source
TGFG: Culture suspensions + L-MRS medium (2% (w/v) glucose) with 0.01% (w/v) free gossypol
