# Supplementary material for: The rumen-derived Lact. mucosae LLK-XR1 exhibited greater free gossypol degradation capacity during solid-state fermentation of cottonseed meal and probiotic potential
Source: BMC Microbiol. 2024 Jan 5;24:15. doi: 10.1186/s12866-023-03156-6 (PMC10768434; doi:10.1186/s12866-023-03156-6)
Supplement: Supplementary file 1 — Supplementary Material 1 [file 12866_2023_3156_MOESM1_ESM.docx]

**Supplementary Materials**

In this study, we obtained 428 records from the Web of Science All databases (time span: 2003.01.01 to 2023.01.01, search strategy: Topic (free gossypol), <https://www.webofscience.com>). The 167 Keywords related to gossypol were analyzed by VOSviewer. All of them were mainly classified into four clusters. The four clusters were about “gossypol”, “cottonseed meal”, “cottonseed”, and “free gossypol”, respectively. From our study, the keywords of livestock types connected around “gossypol” were “bulls”, “cattle”, and “sheep”. The keywords connected around “free gossypol” were “fermentation”, “optimization”, “lactic acid bacteria”, “detoxification”, and “solid-state fermentation”.These literature focused on different tolerance of gossypol between ruminants and monogastric animal issues and determinants of free gossypol degradation.
